# Editorial: Arbuscular Mycorrhizal Fungi: The Bridge Between Plants, Soils, and Humans

**DOI:** 10.3389/fpls.2022.875958

**Published:** 2022-04-04

**Authors:** Sergio Saia, Jan Jansa

**Affiliations:** ^1^Department of Veterinary Science, University of Pisa, Pisa, Italy; ^2^Czech Academy of Sciences, Institute of Microbiology, Prague, Czechia

**Keywords:** beneficial microbes, nutrient limitation, human needs, soil health, plant stress, pathogens, sustainable agro-ecosystem, sustainability

It is assumed that arbuscular mycorrhizal (AM) symbiosis is established between the roots/rhizoids of ca 70% of all plant species, including some of the most important crops, and specialized soil fungi (Brundrett and Tedersoo, 2018). The AM fungi provide a direct interconnection between roots and soil as well as between root systems of different plant individuals belonging to the same or different plant species (Walder et al., 2012; Weremijewicz et al., 2016; Rezáčová et al., 2018b). The AM fungi exert several direct (e.g., enhanced nutrient acquisition, pollutant immobilization/detoxification, plant carbon reallocation, induced pathogen tolerance, signal transfer), and indirect (e.g., photosynthesis stimulation, drought tolerance, soil physical, and microbial conditioning) effects on the plants, with possible consequences to yield and agricultural product quality, multitrophic interaction networks, and soil quality (Kaschuk et al., 2009; Johnson, 2010; Smith et al., 2010; Garg and Chandel, 2011; Rezáčová et al., 2018a). Besides having a finely tuned molecular dialogue with their plant hosts, the fungi also interact with soil microbes fulfilling important ecosystem functions, such as organic nutrient mineralization or stabilization of soil organic matter (Verbruggen et al., 2016; Jiang F. et al., 2021; Rozmoš et al., 2021; Sánchez et al., 2021), and these interactions are further modulated by environmental (soil, climatic, ecosystem management) contexts (Frey-Klett et al., 2007; Hoeksema et al., 2010).

The goal of this Research Topic was to provide an illustration of the range of different views on the functioning of AM symbiosis in natural and anthropogenic systems, to promote mechanistic understanding of the formation, extent, and dynamics of the AM fungal hyphal networks and associated microbes in the soil and to add to the mounting knowledge on the feedbacks between human activities and ecosystem functioning, with a particular focus on the role of AM fungal symbiosis across various plant species/genotypes, soil types, and climatic regions.

We received 30 manuscripts dedicated to the various aspects of mycorrhizal symbioses specified above, out of which 11 were eventually accepted for including in this collection. These publications deal with plant-AM fungi interactions (and occasionally also with other mycorrhizal types, Table 1) and their role in satisfying various human needs. They include “review/meta-analyses” (three articles), “genotype, genetic, and omics aspects” (five articles), “abiotic stress and plant nutrition aspects” (nine articles); “monocots” (two articles); “tree species” (three articles); and works performed under “field conditions” (eight articles). Particularly, we are happy to cover the latter aspect quite broadly, of which the studies are usually underrepresented in the literature compared to the research carried out under controlled conditions (Lekberg and Koide, 2005; Kaschuk et al., 2010; Pellegrino et al., 2015; Zhang et al., 2019; Jiang S. et al., 2021; Qiu et al., 2022; Salomon et al., 2022).

**Table 1:** Synopsis of the articles published in the collections and number of topics covered.

**Title (Citation)**	**Review or metanalysis**	**Genotype, genetic, and omic**	**Abiotic stress and nutrition**	**Monocots**	**Trees**	**Field conditions**
Understanding Multilevel Selection May Facilitate Management of Arbuscular Mycorrhizae in Sustainable Agroecosystems (Johnson and Gibson)	1					1
Nitrogen fertilization increases specific root respiration in ectomycorrhizal but not in arbuscular mycorrhizal plants: a meta-analysis (Bicharanloo et al.)	1		1			1
Potential effects of microplastic on arbuscular mycorrhizal fungi (Leifheit et al.)	1		1			1
Arbuscular mycrorrhizal fungi inoculation and applied water amounts modulate the response of young grapevines to mild water stress in a hyper-arid season (Torres et al.)	0	0	1	0	1	1
Production of organic acids by arbuscular mycorrhizal fungi and their contribution in the mobilization of phosphorus bound to iron oxides (Andrino et al.)	0	0	1	0		0
The phosphate inhibition paradigm: host and fungal genotypes determine arbuscular mycorrhizal fungal colonization and responsiveness to inoculation in cassava with increasing phosphorus supply (Peña Venegas et al.)	0	1	1	0		1
Arbuscular mycorrhizal fungi (*Rhizophagus clarus*) and rhizobacteria (*Bacillus subtilis*) can improve the clonal propagation and development of teak for commercial plantings (Alexandre et al.)	0	0	0	0	1	1
Effects of mycorrhizal colonization on transcriptional expression of the responsive factor JERF3 and stress-responsive genes in banana plantlets in response to combined biotic and abiotic stresses (Rashad et al.)	0	1	2	1	0	0
Stoichiometry of carbon, nitrogen and phosphorus in shrub organs linked closely with mycorrhizal strategy in Northern China (Yang et al.)	0	1	1	0	1	1
Similar arbuscular mycorrhizal fungal communities in 31 durum wheat cultivars (*Triticum turgidum* L. var. durum) under field conditions in Eastern Canada (Stefani et al.)	0	1	0	1	0	1
Arbuscular mycorrhizal fungi improve tolerance of the medicinal plant *Eclipta prostrata* (L.) and induce major changes in polyphenol profiles under salt stresses (Duc et al.)	0	1	1	0	0	0

The results of the currently presented research thus contribute in a relevant way to the long ongoing debate on the use and usefulness of the mycorrhizal technology in agriculture and its prospective as an agronomic strategy for the future (Frossard et al., 2009; Gianinazzi et al., 2010; Ryan and Graham, 2018; Rillig et al., 2019; Ryan et al., 2019; Benami et al., 2020; Brito et al., 2021). We believe that such a multifaceted debate, also happening in other aspects of the crops and/or environmental management (Sadras et al., 2020), is much needed and to a large extent still not reaching a broad consensus, mainly due to the lack of sufficient data and generally valid concepts. Indeed, more detailed information seems to be needed on how different agronomic practices affect indigenous AM fungi in the different soils, how this links to human needs, and how this all is affected by changing environmental context. Besides, novel concepts and demonstrations of their validity are needed in terms of potential manipulation of the systems for the good of the humans and the ecosystems. This collection contributes to reducing such knowledge gaps.

In particular, two excellent perspectives cover the establishment and functions of the AMF symbiosis in sustainable agroecosystems: Johnson and Gibson pointed out that the mycorrhizal role and benefit should be studied and understood while considering a multilevel selection resulting in a local adaption. Along similar lines, Bicharanloo et al. clarified, through a meta-analytical approach, that specific root respiration (SRR), which could be regarded as a proxy for mycorrhizal costs, did not vary among the AM and non-mycorrhizal (NM) plants subjected to different N fertilization levels, in contrast to ectomycorrhizal (ECM) plants. These results are complementary to insightful comparisons between the two different types of mycorrhizal symbiosis across gradients of environmental stoichiometry provided by Yang et al., who found a higher nitrogen concentration in AM-associated compared to the ECM fungi-associated shrub plants, and who also observed opposite trends for phosphorus concentrations, thus suggesting that under C non-limited conditions, such as in the shrub plants, the N demand by AM fungi can stimulate plant growth benefits particularly under higher N availability. Results by Bicharanloo et al. may have thus depended the variability in the C-sink limitations among the plant species included (Gamper et al., 2004). Nonetheless, the results also provide indirect evidence that management of AM fungal activity (either through inoculation or conservation of the indigenous AM fungi) should not be ignored even under intensively fertilized systems, particularly if underrepresented plant/crop species are considered.

Leifheit et al. tackled the possible implication for the AM fungi of one of the understudied factor of global change, namely the soil pollution with microplastics. After reviewing the current knowledge, they elaborate on the priorities for future experimental work focused on understanding the importance of such emerging thread as the microplastics for ecosystem functioning in general and the AM symbiosis in particular.

Other articles included in this collection cover a wealth of aspects relevant to mycorrhizal functioning under various agroecosystems and environmental settings. Stefani et al. studied the recruitment of the native AM fungal communities in contrasting genotypes of durum wheat in Canada, showing that the main environmental filter was actually the plant genotype identity and the traits independent of the root colonization rate. These results are not only demonstrating that different genotypes recruit quite different AM fungi from the same soil pool. Together with previous research (e.g., Jansa et al., 2008; Lendenmann et al., 2011; Veiga et al., 2011; Thonar et al., 2013; Knegt et al., 2016), these results allow speculations about consequences of changing crop genotype (or crop mixture composition) to derive different mycorrhizal benefits in terms of plant nutrition and/or growth, as well as co-existence of the crops with weeds.

Peña Venegas et al. showed that cassava farming in equatorial Africa could benefit from the inoculation with specific genotypes of AM fungi even under high P supply, which has previously been considered detrimental for the development and/or functioning of the AM fungi. These results align nicely with the elegant experiment of Andrino et al., who showed that in tomato, the AM fungi could release low-molecular-weight organic acids that facilitate P uptake from highly P-fixing soil along with other elements, such as Fe. Andrino et al. also pointed that membrane fluidity could vary in the AM fungi supplied with various P sources and this can further affect their P uptake from the soil and delivery to their host plants.

Alexandre et al. worked on teak (*Tectona grandis* L.f.), an important timber species in Brazil, and confirmed that the amendment of clonally propagated plantlets with AM fungi and plant growth-promoting rhizobacteria could significantly improve plant growth and nutrition, thus fostering their field growth between 4.75 and 11.04%. Similarly, Rashad et al. demonstrated mycorrhizal benefits and underlying metabolic pathways in banana plantlets subjected to different abiotic (salinity) and biotic (pathogen) stresses. Along similar lines, mycorrhizal benefits were also elaborated on the medicinal plant *Eclipta prostrata* (Duc et al.) subjected to salinity stress, which seem to have been coupled with modulation of the polyphenol production. Thus, the present results generally confirm previous findings on the ability of the AM fungi to modulate secondary metabolism (Zeng et al., 2013; Lazzara et al., 2017; Saia et al., 2019) of a range of medicinal and other plants, despite the remaining uncertainty in the mycorrhizal effects on the volatile compounds composition (Saia et al., 2021).

Somewhat contrasting with the previous research, Torres et al. found that in grapevines, the AM fungi affected more the secondary compound production under contrasting water availability regimes than the leaf mineral content (indicative of direct nutritional effects). These differences between the results of Torres et al. and the previously cited works could possibly be explained by differential nutrient reserves accumulated in the plants before onset of the stresses, which could potentially buffer the stress impact on plants and promote apparition of other than nutritional mycorrhizal benefit.

The majority of the studies thus aimed at elucidating physiological mechanism of the abiotic stress responses in AM plants. All the studies contributed to the debate on greater utilization of mycorrhizal symbiosis in agronomy and forestry practices, either as planned interventions or as a collateral effect. Importantly, 8 out of the 11 studies has been conducted, or dealing with, field conditions. We believe the integration of these results contributed to create useful resource for our peers and students. We would like to thank all contributors for their work and patience in these uneasy COVID times and to the Frontiers staff for their relentless support and assistance for making this to happen.

## Author Contributions

Both authors equally contributed to this editorial and the special issue management. All authors contributed to the article and approved the submitted version.

## Conflict of Interest

The authors declare that the research was conducted in the absence of any commercial or financial relationships that could be construed as a potential conflict of interest.

## Publisher's Note

All claims expressed in this article are solely those of the authors and do not necessarily represent those of their affiliated organizations, or those of the publisher, the editors and the reviewers. Any product that may be evaluated in this article, or claim that may be made by its manufacturer, is not guaranteed or endorsed by the publisher.
